# Differential activation of hepatic stellate cells by *Echinococcus multilocularis* and *Echinococcus granulosus* protoscoleces

**DOI:** 10.1007/s00436-026-08681-7

**Published:** 2026-05-18

**Authors:** Emad Shamsan, Liu Chuanchuan, Fan Haining

**Affiliations:** 1 College of Clinical Medicine, Qinghai University, Xining, 810001 China; 2 College of Medical Science, Taiz University, Taiz, Yemen; 3 Qinghai University Affiliated Hospital, Xining, 810001 China

**Keywords:** *E. multilocularis*, *E. granulosus*, Hepatic stellate cells, Protoscoleces, Collagen-I, α-SMA, Osteopontin, TNF-α

## Abstract

Echinococcosis, caused by *Echinococcus multilocularis* (Em) and *Echinococcus granulosus* (Eg), induces distinct patterns of liver fibrosis. Understanding how protoscoleces (PSCs) from these species interact with hepatic stellate cells (HSCs) is crucial to elucidating the mechanisms of fibrogenesis. In this study, HSC-LX2 cells were co-cultured with Em and Eg PSCs. ELISA was used to quantify collagen-I, α-smooth muscle actin (α-SMA), osteopontin (OPN), and TNF-α levels, while transmission electron microscopy (TEM) examined ultrastructural changes. Both *E. multilocularis*- and *E. granulosus*-derived PSCs significantly increased extracellular matrix (ECM) protein expression, but EmPSCs induced earlier and stronger responses, accompanied by more pronounced structural alterations in HSCs. These findings demonstrate species-specific HSC activation and provide insight into the differential fibrogenic potential of *E. multilocularis* and *E. granulosus*.

## Introduction

*Echinococcus multilocularis* and *Echinococcus granulosus* are parasitic tapeworms responsible for alveolar echinococcosis (AE) and cystic echinococcosis (CE), respectively. Both parasites have complex life cycles involving canids as definitive hosts and herbivores or humans as intermediate hosts, with larval stages primarily developing in the liver and forming characteristic cystic lesions (Hidalgo et al. 2021, WHO 2025, Wen et al. 2019, Deplazes et al. 2017).

HSCs are the principal effector cells in liver fibrogenesis. Upon activation by liver injury or pathogenic stimuli, HSCs undergo transdifferentiation into myofibroblast-like cells, characterized by increased production of ECM proteins such as collagen I and α-SMA, as well as the secretion of pro-fibrotic mediators including OPN (Chen et al. 2018, Cao et al. 2021). Parasitic infections are increasingly recognized as important modulators of hepatic fibrogenesis through their effects on HSCs. In particular, studies on *Schistosoma species* have shown that parasite-derived components can directly stimulate HSCs in vitro, inducing phenotypic activation and the release of inflammatory and fibrogenic mediators (Müller et al. 2024). These effects are further supported by mechanistic evidence demonstrating that *Schistosoma japonicum*-induced fibrosis is regulated through pathways such as galectin-mediated signaling and the Hippo/YAP axis, which control the expression of key ECM proteins including α-SMA and collagen (Huang et al. 2024, Xuan et al. 2025). Notably, the patterns of periparasitic fibrosis differ between AE and CE. AE is characterized by progressive, infiltrative lesions associated with extensive fibrosis, whereas CE typically presents as well-defined cysts surrounded by relatively limited fibrotic tissue (Niu et al. 2019). These differences suggest species-specific host–parasite interactions and distinct mechanisms of HSC activation.

Although previous studies have examined HSC activation in response to individual parasite-derived components, including hydatid fluid and PSCs (Cao et al. 2021, Zhang et al. 2016), direct comparative analyses of the fibrogenic effects of Em and EgPSCs remain limited. Therefore, this study aimed to compare the activation of HSC-LX2 cells induced by EmPSCs and EgPSCs by assessing key fibrogenic markers and associated cellular changes, providing insight into species-specific mechanisms underlying echinococcosis-associated fibrosis.

## Materials and methods

### Parasite material and culture conditions

EgPSCs were aseptically collected from hepatic hydatid cysts in naturally infected sheep from a slaughterhouse in Xining, Qinghai, China. EmPSCs were harvested from the livers of experimentally infected Mongolian gerbils (*Meriones unguiculatus*) following a six-month infection period. All PSCs were extensively washed in phosphate-buffered saline (PBS, pH 7.4) containing 1000 U/mL penicillin and 1000 µg/mL streptomycin. The viability of PSCs was assessed using the eosin exclusion test prior to co-culture (Aboelsoued et al. 2024), and was confirmed to be > 95%. PSCs were then maintained in vitro in a culture medium, as previously described (Cao et al. 2021), at 37 °C in a 5% CO₂ atmosphere.

## Cell culture and co-culture conditions

HSC-LX2 was obtained from Beijing University of Biological Sciences (Beijing, China). Cells were cultured in Dulbecco’s Modified Eagle Medium (DMEM) supplemented with 10% fetal bovine serum (FBS), penicillin (1000 U/mL), and streptomycin (1000 µg/mL) at 37 °C in a 5% CO₂ incubator.

For co-culture experiments, HSC-LX2 cells were seeded in 60-mm culture plates at a density of 1.5 × 10⁵ cells per plate and allowed to adhere overnight. The following experimental groups were established:

- HSC + EmPSCs: HSC-LX2 cells co-cultured with *E. multilocularis* PSCs.
- HSC + EgPSCs: HSC-LX2 cells co-cultured with *E. granulosus* PSCs.
- HSC Control: HSC-LX2 cells cultured alone.

PSCs were added to the respective plates at a ratio of 1 PSC to 200 HSCs (1:200). Co-cultures were maintained for 48, 72, and 96 h prior to supernatant and cell collection.

## Enzyme-linked immunosorbent assay

Following the co-culture periods, the supernatant from each group was collected and centrifuged at 1000×g for 20 min to remove cellular debris. The levels of secreted human Col-I, α-SMA, OPN, and TNF-α in the supernatant were quantified using specific commercial sandwich ELISA kits (Cloud-Clone Corp., USA), according to the manufacturer’s instructions. The optical density (OD) of each well was measured at 450 nm using a microplate reader, and protein concentrations were determined by interpolation from the standard curve.

## Transmission electron microscopy

After 48, 72 and 96 h of co-culture, HSC-LX2 cells were washed with PBS, detached using trypsin, and pelleted by centrifugation. The cell pellets were fixed in 2.5% glutaraldehyde in 0.1 M phosphate buffer (pH 7.4) at 4 °C for a minimum of 4 h. Subsequent processing included post-fixation in 1% osmium tetroxide, dehydration through a graded ethanol series, and embedding in epoxy resin. Ultrathin Sects.  (70–90 nm) were cut using an ultramicrotome, stained with uranyl acetate and lead citrate, and examined under a Hitachi HT7700 transmission electron microscope operated at 80 kV.

### Statistical analysis

All quantitative data are presented as the mean ± standard deviation (SD) from at least three independent experiments. Statistical analysis was performed using GraphPad Prism software (Version 10). Differences between multiple groups were analyzed by one-way analysis of variance (ANOVA) followed by Dunnett’s post-hoc test for comparisons against the control group. A *p*-value of less than 0.05 was considered statistically significant.

## Results

### ECM protein expression by ELISA

**Fig. 1:**
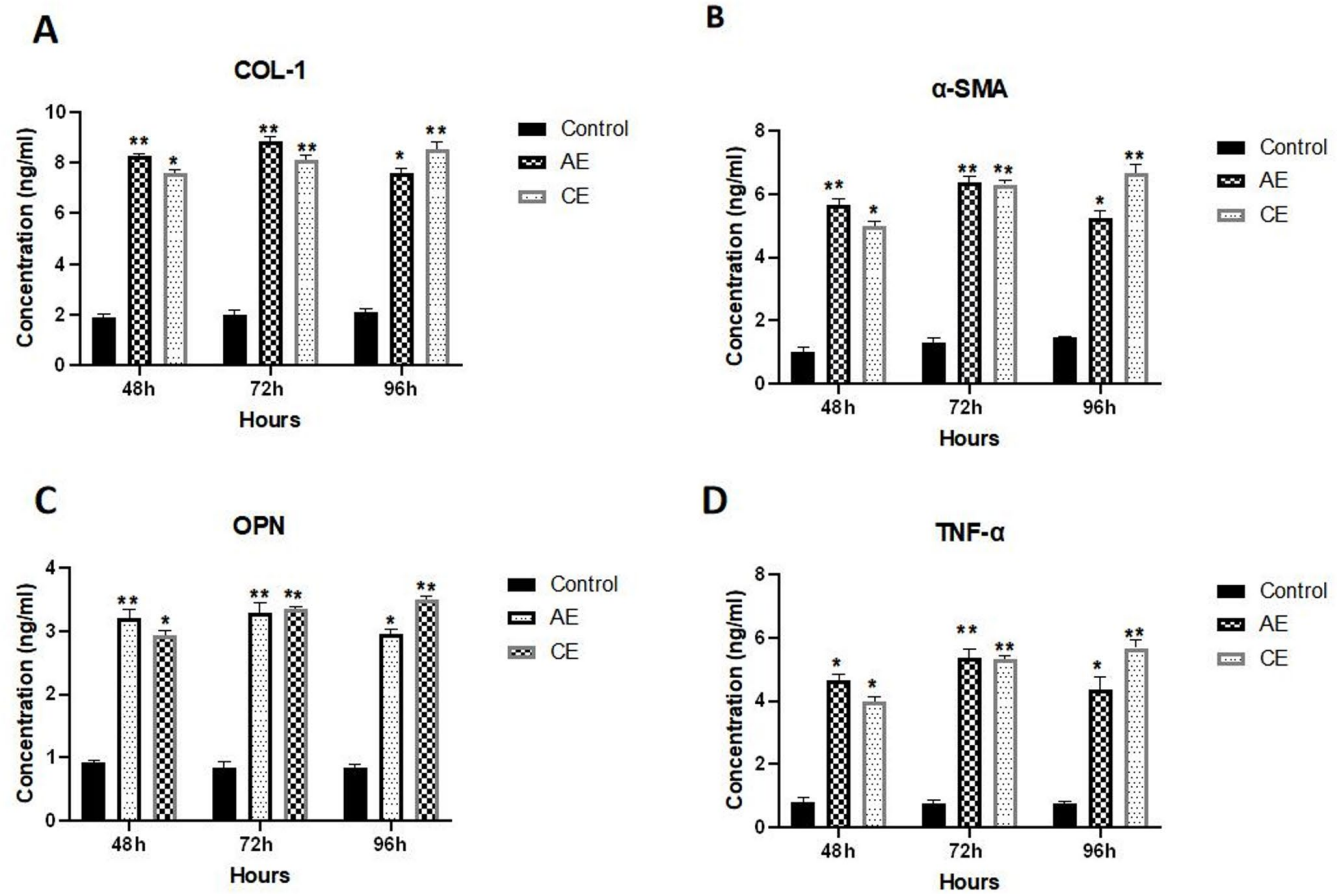
ELISA results showing collagen-I, α-SMA, OPN, and TNF-α levels in HSCs exposed to EmPSCs, EgPSCs, and control. EmPSCs induce rapid early peaks; EgPSCs show sustained increases

Both EmPSCs and EgPSCs PSCs significantly upregulated coll-I, α-SMA, OPN, and TNF-α in HSCs compared to controls (Fig. 1). EmPSCs-treated HSCs exhibited a rapid increase, peaking at 72 hours, followed by a slight decline at 96 h, likely due to cytotoxic effects. EgPSCs-treated HSCs showed a gradual and sustained increase over 96 hours.

## Ultrastructural changes by TEM

**Fig. 2:**
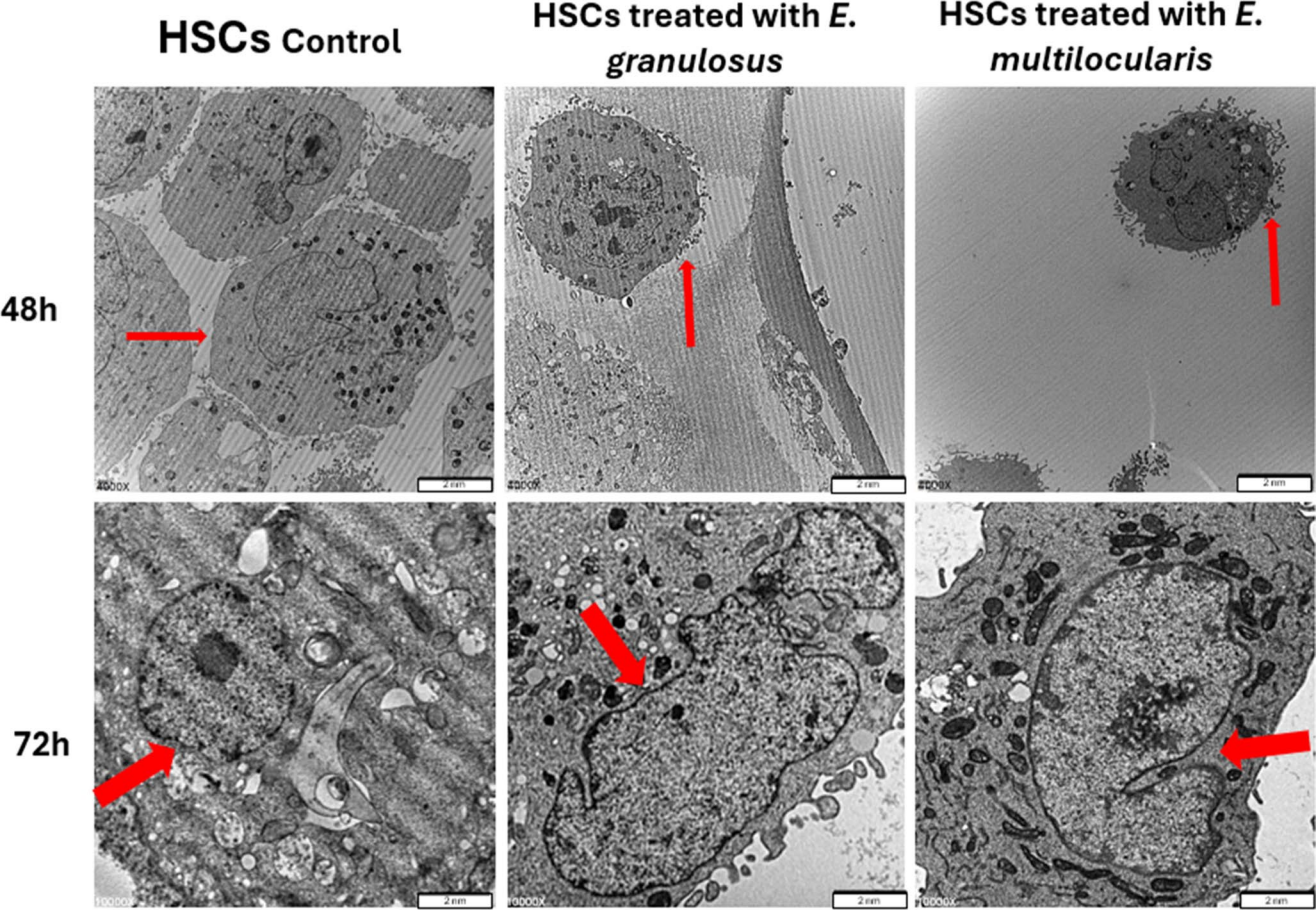
The figure illustrates microvilli on (HSCs after 48 hours, with clear visibility noted particularly in those treated with EmPSCs (arrow), contrasting with the comparatively fewer microvilli observed in HSCs treated with EgPSCs and the absence of microvilli in the control group. After 72 hours, irregular and distorted nuclei are evident, especially in HSCs treated with EmPSCs

**Fig. 3:**
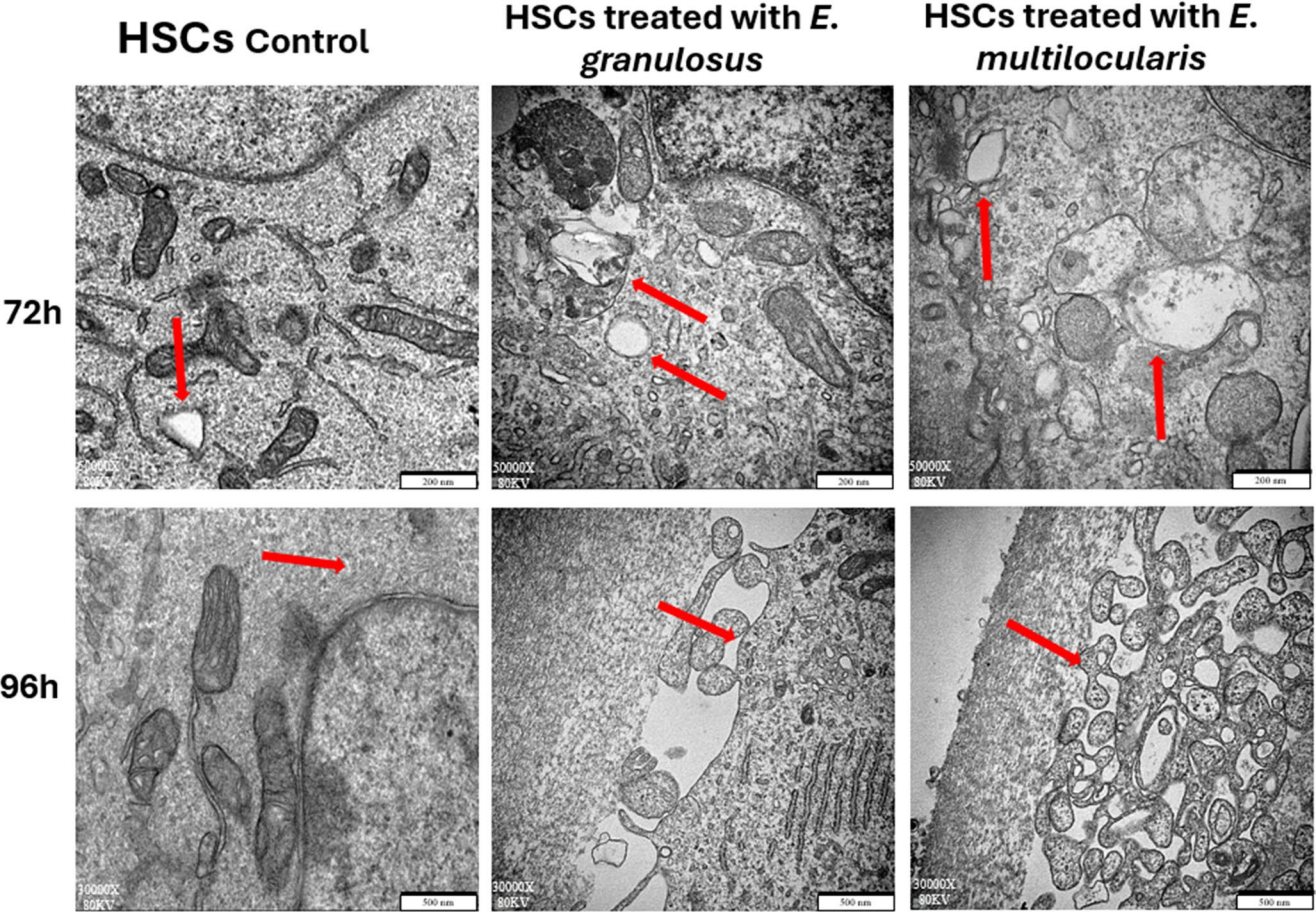
At 72 hours, there is a noticeable enlargement of oval lipid droplets, particularly evident in HSCs treated with EmPSCs, surpassing those treated with EgPSCs. By 96 hours, cytoplasmic content exhibits signs of degradation, with a pronounced decrease observed, especially in cells treated with EmPSCs

TEM analysis revealed distinct, species-specific ultrastructural pathologies in HSCs (Figs. 2 and 3). HSCs co-cultured with EgPSCs displayed morphological features associated with apoptosis, including the presence of apoptotic bodies and irregular, distorted nuclei (Fig. 2). In stark contrast, HSCs exposed to EmPSCs exhibited severe cytoplasmic disruption. This was characterized by a massive enlargement of intracellular lipid droplets evident at 72 h, which progressed to widespread cytoplasmic degradation and organelle damage by 96 h (Figs. 2 and 3).

## Discussion

Our findings demonstrate that PSCs from Em and Eg activate HSCs, increasing ECM expression and inducing ultrastructural changes. EmPSCs elicited a stronger and more rapid response, consistent with the aggressive fibrosis observed in AE, whereas EgPSCs induced slower, sustained activation, reflecting CE.

The villi-like protrusions observed on HSCs likely represent cytoskeleton-associated membrane remodeling during activation. Such structural changes are consistent with actin cytoskeleton reorganization and the formation of stress fibers described in activated HSCs (Łotowska et al. 2023, Cui et al. 2014). Lipid droplets may facilitate HSC activation and energy metabolism, consistent with TGF-β-induced activation in stem cell-derived HSCs (Wilhelmsen et al. 2024) and metabolic signaling from lipotoxic hepatocyte-derived vesicles (Li et al. 2024). Comparable alterations have been reported in other parasitic infections, including *Schistosoma spp*., providing broader context (Bülow et al. 2023, Carson et al. 2018, Chen et al. 2016).

Ultrastructural imaging showed substantially greater HSC damage following *E. multilocularis* exposure, suggesting species-specific injury mechanisms. These observations align with recent work showing HSC activation and ECM accumulation in* Echinococcus* infections (Cao et al. 2021, Tian et al. 2021). Understanding these mechanisms may inform targeted antifibrotic strategies and improve insight into the pathogenesis of echinococcosis.

## Conclusion

EmPSCs induce a stronger and more rapid activation of HSCs compared to EgPSCs, both in ECM protein expression and structural alterations. This comparative analysis highlights species-specific host-parasite interactions that contribute to differential liver fibrosis in AE and CE. These findings provide a foundation for further mechanistic studies and potential therapeutic targeting.

## Data Availability

The data supporting the findings of this study are available from the corresponding author upon reasonable request.

## Abbreviations

AE: Alveolar echinococcosis
CE: Cystic echinococcosis
α-SMA: Alpha–smooth muscle actin
Col–I: Collagen–I
DMEM: Dulbecco’s Modified Eagle Medium
ECM: Extracellular matrix
ELISA: Enzyme–linked immunosorbent assay
HSC: Hepatic stellate cells
OPN: Osteopontin
TNF-α: Tumor Necrosis Factor–alpha
PSC: Protoscoleces
TEM: Transmission Electron Microscopy
EmPSCs: *E. multilocularis* protoscoleces
EgPSCs: *E. granulosus* protoscoleces
